# Usual choline and betaine dietary intake and incident coronary heart disease: the Atherosclerosis Risk in Communities (ARIC) Study

**DOI:** 10.1186/1471-2261-7-20

**Published:** 2007-07-13

**Authors:** Aurelian Bidulescu, Lloyd E Chambless, Anna Maria Siega-Riz, Steven H Zeisel, Gerardo Heiss

**Affiliations:** 1 Department of Epidemiology, School of Public Health, University of North Carolina, Chapel Hill, NC, USA; 2 Department of Biostatistics, School of Public Health, University of North Carolina, Chapel Hill, NC, USA; 3 Department of Nutrition, School of Public Health, University of North Carolina, Chapel Hill, NC, USA

## Abstract

**Background:**

Low dietary intake of the essential nutrient choline and its metabolite betaine may increase atherogenesis both through effects on homocysteine methylation pathways as well as through choline's antioxidants properties. Nutrient values for many common foods for choline and betaine have recently become available in the U.S. nutrient composition database. Our objective was to assess the association of dietary intake of choline and betaine with incident coronary heart disease (CHD), adjusting for dietary intake measurement error.

**Methods:**

We conducted a prospective investigation of the relation between usual intake of choline and betaine with the risk of CHD in 14,430 middle-aged men and women of the biethnic Atherosclerosis Risk in Communities study. A semi-quantitative food frequency questionnaire was used to assess nutrient intake. Proportional hazard regression models were used to calculate the risk of incident CHD. A regression calibration method was used to adjust for measurement error.

**Results:**

During an average 14 years of follow-up (1987–2002), 1,072 incident CHD events were documented. Compared with the lowest quartile of intake, incident CHD risk was slightly and non-significantly higher in the highest quartile of choline and choline plus betaine, HR = 1.22 (0.91, 1.64) and HR = 1.14 (0.85, 1.53), controlling for age, sex, education, total energy intake, dietary intakes of folate, methionine and vitamin B_6_. No association was found between dietary choline intake and incident CHD when correcting for measurement error.

**Conclusion:**

Higher intakes of choline and betaine were not protective for incident CHD. Similar investigations in other populations are of interest.

## Background

The essential nutrient choline, its metabolite betaine, as well as folate and methionine are all metabolically interrelated by transmethylation pathways [1-4]. Through under-methylation of DNA, low dietary intakes of choline and betaine alter the epigenetic regulation for a series of genes reported to accelerate the atherogenic process [5,6]. Like folate, choline is involved in the methylation of homocysteine (a putative cardiovascular risk factor) to methionine through a betaine-dependent pathway. When folate availability diminishes there is an increased demand for betaine as a methyl donor [7]. Conversely, when choline availability is decreased the demand for folate is increased [8]. Because of the interrelationship of folate and choline pathways, both nutrients should be considered in epidemiological studies assessing the relationship between dietary intake of these compounds and cardiovascular disease.

Until recently analysis of choline intake was not possible because the choline content of most foods had not been measured accurately [9,10] and thus far only three observational studies have been published of dietary intake of choline and betaine [11-13]. We investigated the hypothesized association of a low dietary intake of choline and betaine with incident coronary heart disease (CHD) in a large middle-aged biracial cohort of men and women sampled from four U.S. locales. We further investigated whether the estimated risk of CHD varies by intake of folate or methionine, by menopausal status, sex or race. We contrasted the results with those obtained using a calibration method to adjust for known measurement error in the assessment of four interrelated nutrients: choline, folate, methionine and total energy intake.

## Methods

The study was conducted in the cohort component of the Atherosclerosis Risk in Communities (ARIC) Study, a prospective observational biracial follow-up of 15,792 men and women between the ages of 45 and 64, recruited from Forsyth County, NC, Jackson, MS, suburbs of Minneapolis, MN, and Washington County, MD [14]. Institutional review board approval was obtained by each participating field center and the coordinating center. Written informed consent was obtained from each study participant. The analyses excluded cohort members who had CHD at baseline (n = 766), race other than white and African-American (n = 48), missing dietary information for either folate or methionine (n = 8), and extreme reported caloric intake values (below 500 kcal for women and 700 kcal for men, and above 3,500 for women and 4,500 for men, corresponding to the 3^rd ^and 97^th ^percentiles of the data distribution; n = 540). Prevalent CHD was defined as evidence of a prior myocardial infarction (MI) by electrocardiogram readings taken during the baseline clinic visit, self-reported physician diagnosis of MI, or self-reported cardiovascular surgery/coronary angioplasty. After applying these exclusions, 14,430 individuals remained for analysis.

The ingested choline and betaine was quantified, during the baseline visit (1987–1989), with a 66-item version of the Willett semi-quantitative food frequency questionnaire, FFQ [15]. The participants were asked how often, on average, they had consumed listed food items during the previous year. Nine frequency responses were listed ranging from more than six per day to almost never. We calculated daily servings by converting the consumption frequency to servings per day. Dietary choline and betaine were estimated as the sum of daily intakes, using a choline and betaine database composed with the U.S. Department of Agriculture (USDA) choline and betaine content in common foods database, database that contains 207 food items [10].

Incident non-fatal MI and fatal CHD were ascertained, validated and classified following the standardized ARIC cohort and community surveillance protocol [16]. We considered the intakes of choline, and choline plus betaine, in multivariable models. We adjusted for total energy intake as a continuous variable after evaluating that adjustment using the residual method [17] produced similar results. The following confounding variables were also included in the models: age, sex, education, dietary folate, methionine, B_6 _vitamin and cholesterol, race, diabetes, ARIC center, menopausal status (reported cessation of menses), and a series of CHD risk factors such as smoking, hypertension, body mass index (BMI) and family history of CHD. Using a likelihood ratio test we assessed the effect measure modification of dietary folate intake, sex, menopausal status, race, education, ARIC center and alcohol intake. Due to women's capacity to form the choline moiety de novo the amount of choline necessary in the daily diet is influenced by sex and menopausal status. Alcohol intake, a known folate antagonist, may plausibly increase the requirement for folate intake. Folate and alcohol were categorized with a cutoff point at the lowest and the highest quartile, respectively. We calculated hazard ratios (HR), per quartiles of dietary intake, using Cox proportional hazard regression. Verification of the proportional hazard assumptions was assessed using plots of the log(-log) survival curves. We contrasted the results with those obtained using the procedure described below to correct for measurement error. Statistical analyses were conducted using SAS software [18]. All p-values were two-tailed.

We applied regression calibration [19-21] to correct for measurement error in the following independent variables: choline (choline plus betaine), folate, methionine and total energy intake. To enable this adjustment we assessed the reliability of the dietary instrument in a random sample of 1,004 subjects whose dietary intake was measured three years after the ARIC baseline. From each field center an equal number of participants were selected. The dietary form was administered in the identical manner as was done during the ARIC baseline examination. The intraindividual variability was calculated and the correlation between measures made at repeat visits, the reliability coefficient, was estimated using mixed models regression [22]. The reliability coefficients for the nutrients of interest used for the adjustment procedure were 0.50 for choline (0.50 for choline plus betaine), 0.53 for folate, 0.48 for methionine and 0.43 for caloric intake. The general estimator for the measurement error model was applied to the longitudinal analysis of dietary choline with incident CHD. We used a bootstrapping technique to estimate the variance of beta coefficients obtained in the final longitudinal analysis corrected for measurement error. We repeated the bootstrap sampling one thousand times.

## Results

Among the 14,430 participants in this study, the median intake of choline was 302 mg/day in men, 271 mg/day in women, 286 mg/day in whites and 274 mg/day in blacks. For betaine the median intakes were 101 mg/day in men, 89 mg/day in women, 95 mg/day in whites and 91 mg/day in blacks. The distribution of the main baseline characteristics of the study population according to gender is presented in Table 1. The correlations between the interrelated nutrients choline, folate and methionine were as follows: 0.35 for choline-folate, 0.45 for choline-methionine and 0.55 for folate-methionine. In our population, the top five contributors of choline intake were red meat – side dish (11.4%), eggs (11.1%), red meat – main dish (10.4%), low fat milk (8.3%) and chicken without skin (8.3%). The top five contributors of betaine were dark bread (24.9%), white bread (18.1%), spinach (10.4%), cold breakfast cereals (8.2%) and pasta (4.7%). The top ten food items (and the quantity per serving) on the FFQ that contribute to the intake of these two micronutrients in the ARIC population are presented in Table 2.

**Table 1:** Distribution of baseline characteristics of the ARIC Study population (N = 14,430) according to gender.

**Characteristic**	**Gender**	
	
	**Men**	**Women**
Age, y*	54.4 (5.76)	53.7 (5.71)
African American, %	23.0	29.9^†^
Diabetes, %	8.6	9.6
Current smoker, %	27.5	24.6^†^
Body mass index, kg/m^2^*	27.4 (4.15)	27.8 (6.10)
Choline intake, mg/day*	332.1 (124.7)	294.2 (111.9)
Betaine intake, mg/day*	118.1 (55.4)	102.4 (47.1)
Alcohol intake, g/day*	10.16 (17.83)	2.90 (7.21)
Dietary cholesterol, mg/day*	278.2 (139.4)	230.2 (112.0)
Dietary methionine, g/day*	1.74 (0.66)	1.63 (0.66)
Dietary fiber, g/day*	17.3 (7.94)	16.9 (8.02)
Dietary folate, μg/day*	234.4 (102.4)	221.5 (100.2)
Dietary saturated fatty acid, g/day*	24.2 (10.6)	19.9 (9.2)

* Numbers presented are mean (SD).

^† ^p < 0.001 for chi-square test comparing the two genders.

**Table 2:** Top ten food items on the FFQ that contribute to the intake of choline and betaine among 14,430 participants in the ARIC Study.

**Choline**			
**Ranking**	**Food group**	**% of nutrient**	**Quantity per serving (mg)**

1	Read meat – side dish	11.4	163.5
2	Eggs	11.1	110.4
3	Red meat – main dish	10.4	130.1
4	Low fat milk	8.3	40.7
5	Chicken without skin	8.3	111.6
6	Chicken or turkey (with skin)	4.0	92.8
7	Coffee	3.5	6.2
8	Fish (cod, perch, catfish)	3.3	94.8
9	Potatoes, mashed or baked	3.3	32.9
10	Whole milk	2.6	34.6

**Betaine**			

**Ranking**	**Food group**	**% of nutrient**	**Quantity per serving (mg)**

1	Dark bread	24.9	34.4
2	White bread	18.1	25.5
3	Spinach	10.4	72.9
4	Cold breakfast cereals	8.2	21.6
5	Pasta	4.7	34.9
6	Cookies	3.6	11.9
7	Coffee	3.1	1.9
8	Hamburgers	2.5	13.8
9	Biscuits or cornbread	2.4	10.9
10	Donut	2.2	25.3

Over an average of 14 years of follow-up (1987–2002), 1072 validated incident CHD events occurred. Neither higher intakes of dietary choline nor betaine were significantly associated with incident CHD. Compared with the lowest quartile of intake, incident CHD risk was 22% higher [HR = 1.22 (0.91, 1.64)] and 14% higher [HR = 1.14 (0.85, 1.53)] in the highest quartile of choline and choline plus betaine respectively, controlling for age, sex, education, total energy intake, and dietary intakes of folate, methionine and vitamin B_6 _(Table 3). Further adjustment for race, diabetes status, ARIC field center and dietary cholesterol, vitamins B_12 _and B_2_, as well as for other CHD risk factors, such as obesity (defined as a body mass index, BMI, higher than 30), hypertension, smoking status and estimated family history of CHD, produced similar risk estimates.

**Table 3:** Hazard rate ratios (and 95% CI) for CHD across quartiles of dietary intakes among 14,430 participants in the ARIC Study.

	**Quartile (Q) of dietary intake**			
	**Q1**	**Q2**	**Q3**	**Q4**
	(N = 3607)	(N = 3608)	(N = 3608)	(N = 3607)
***Choline***	*<217 mg/d*	*217–283 mg/d*	*283–363 mg/d*	*>363 mg/d*
Model #1*	Referent	0.89 (0.73, 1.08)	1.11 (0.90, 1.38)	1.22 (0.91, 1.64)
Model #2^†^	Referent	0.84 (0.69, 1.03)	1.03 (0.82, 1.29)	1.05 (0.76, 1.45)
Model #3^‡^	Referent	0.93 (0.76, 1.13)	1.10 (0.87, 1.37)	1.09 (0.79, 1.50)
***Total choline***	*<298 mg/d*	*298–384 mg/d*	*384–486 mg/d*	*>486 mg/d*
Model #1	Referent	0.91 (0.75, 1.10)	1.07 (0.86, 1.33)	1.14 (0.85, 1.53)
Model #2	Referent	0.87 (0.72, 1.05)	1.01 (0.81, 1.26)	0.99 (0.73, 1.35)
Model #3^‡^	Referent	0.86 (0.67, 1.11)	1.21 (0.97, 1.52)	1.14 (0.83, 1.56)

*In models #1, adjustment was made for age, sex, education, total energy intake, and dietary folate, methionine and vitamin B_6_.

^†^In models #2, adjustment was made for all of the above plus race, diabetes status, ARIC field center, menopausal status and dietary cholesterol.

^‡^In models #3, adjustment was made for all of the variables listed in models #2 plus dietary intake of saturated fatty acids, animal fat, dietary fiber and animal protein.

Because eggs, milk and green vegetables are important contributors to choline and betaine intake, we performed sensitivity-type analyses to further adjust for intake of saturated fatty acids, animal fat, dietary fiber and animal protein. The hazard ratios across the quartiles of choline and total choline intakes (considering the same lowest quartile as referent, and adjusting for all confounders considered in models #2, Table 3, namely age, sex, education, total energy intake, dietary intakes of folate, methionine, vitamin B6, race, diabetes status, ARIC field center, menopausal status and dietary cholesterol) remained practically unchanged, suggesting that the higher risk associated with the highest intake is not due to confounding from uncontrolled dietary factors (Table 3).

No effect measure modification was detected by sex, menopausal status, race, ARIC center, education or folate intake analyzed as a continuous variable. Correction for measurement error in the dietary intake of choline and related nutrients, using models containing continuous nutrient variables, provided similar results. For choline, the hazard ratio for those in the highest quartile, compared with those in the lowest, was 1.34 (0.98, 1.85). When using the bootstrapping procedure and obtained the 95% confidence interval for the corrected choline regression coefficient, the risk estimate remained non-significant.

To examine the effect of folate fortification that was mandated after January 1998 we conducted a supplemental analysis with exclusion of events that occurred prior to that date. For choline and for choline plus betaine the risk estimates were weakened and still not significant. Specifically, compared with the lowest quartile, the hazard ratios for choline were 0.80 (0.62, 1.04), 1.01 (0.76, 1.36) and 0.97 (0.64, 1.47) for the second, the third and the fourth quartile of the dietary intake, respectively. For choline plus betaine, the risks of incident CHD by quartiles were 0.86 (0.67, 1.11), 0.98 (0.73, 1.30) and 0.90 (0.61, 1.35), respectively.

In order to use the repeated measurement of dietary intake completed during visit 3 (1993–1995) in all ARIC participants, we did an additional analysis in which we investigated the risk of incident CHD among participants which did not develop this outcome before this visit. For both choline and for choline plus betaine the risk estimates remained similar. Specifically, compared with the lowest quartile, the hazard ratios for choline were 1.05 (0.81, 1.37), 0.94 (0.70, 1.30) and 1.09 (0.74, 1.59) for the second, the third and the fourth quartile of the dietary intake, respectively. For choline plus betaine, the risks of incident CHD by quartiles were 1.23 (0.94, 1.61), 1.22 (0.89, 1.66) and 1.50 (1.00, 2.26), respectively. We adjusted for all the variables included in the models #3 (table 3).

## Discussion

Over 14 years of follow-up of this large prospective biracial cohort of men and women we did not find a significant association between dietary intake of choline (or choline plus betaine) and the risk of incident CHD, whether or not correction for measurement error was applied. Statistical control for the potential effect of dietary folate, dietary methionine and other covariates did not substantially influence the risk estimates for either choline or choline and betaine. Further, we found no evidence of a monotonic relation with incident CHD across quartiles of dietary intakes of choline.

**Figure 1:**
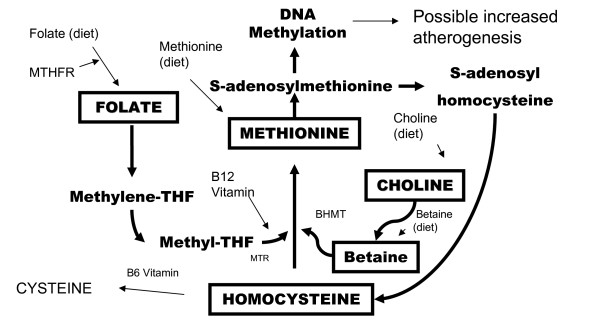
**Metabolism of homocysteine and the remethylationto methionine by the alternative folate and betaine pathways**. THF: tetrahydrofolate; MTHFR: methylentetrahydrofolate reductase; BHMT: betaine-homocysteine methyltransferase; MTR: methionine synthase reductase. Note: in boxes – choline, betaine, folate and methionine concentrations in plasma

Choline, an essential nutrient for humans [23], is found in several compounds that are methyl-donors. Supplementation in the dietary intake range of betaine, a methyl-donor continuously produced from choline [24], leads to immediate and long term lowering of plasma homocysteine [25], a putative CHD risk factor [26]. Homocysteine, a sulfur aminoacid whose metabolism has a direct cytotoxicity effect on vascular endothelium [27], stands at the intersection of two pathways [28]. One catalyzes the synthesis of the amino acid cysteine and the other remethylation to form methionine, a process that requires folate and vitamin B_12_. In an alternative reaction, betaine, the oxidative by-product of choline, serves as a donor of methyl groups to homocysteine to form methionine [29]. Thus, the two metabolic pathways provide alternate mechanisms for removal of homocysteine as shown in Figure 1. The increase in blood homocysteine after a methionine load [3] and consequent vascular cytotoxicity, or the aberrant methylation produced by a low plasma choline and plasma betaine with possible increased atherogenesis [5,6], provide the putative mechanisms that could explain an increase in CHD risk. Alternatively, the intrinsec antioxidant properties of choline [30] could decrease the endothelial dysfunction and the risk for occlusive vascular events in those with a relatively higher intake of this nutrient.

Whereas there is research suggestive of an inverse association between dietary folate and incident CHD [31], the extant literature on dietary choline is small [11-13]; the current study is the first to explore choline as well as betaine in relationship with cardiovascular outcomes in a biracial cohort of both men and women. No relationship was also found recently between dietary intake of choline and betaine and cardiovascular events in a cohort of postmenopausal women enrolled in the Dutch EPIC cohort [13]. Until recently it was not possible to calculate dietary choline intake in humans and there are no nationally representative estimates of this intake from food [23] because the choline content of foods had not been included in major nutrient databases. Food choline data were unreliable due to older, imprecise assay procedures. As a consequence an estimated Average Requirement (EAR) for choline remains to be established. The proposed adequate intake (AI) for choline was set at 550 mg/day for men and 425 mg/day for women [32]. It is unknown whether intakes of choline in the U.S. meet the AI. In the ARIC cohort, the median and 75^th ^percentiles of choline intake were 284 and 367 mg/day, respectively (unpublished results). Only 6% of men and 11% of women had an intake of choline above that proposed as the AI for this population. Choline intake was associated with sex, race and menopausal status.

Repeat measurement mixed modeling permits the estimation of measurement reliability, which in turn permits adjustment for measurement error such as through a regression calibration [19-21]. One approach is to replace the observed values of the variables measured with error (in our case, the nutrients of interest) with multivariate Stein estimates of the true values, conditional on the values of the variables measured without error and the observed values of the variables measured with error [33]. Because the risk estimates relating nutrient intake to CHD were very close to the null, little change in the hazard ratio was observed when measurement error correction was applied.

An explanation for the slightly increased CHD risk toward the higher end of choline intake distribution, opposite to what we initially expected, could be that choline is required for normal secretion of very low density lipoprotein from liver; perhaps provision of choline mobilizes cholesterol from hepatic stores into the vascular pool permitting deposition in atheromas [33]. A higher intake of choline and betaine, which increases the methylation potential of methionine, may result in a change of the cell phenotype that also promotes the development of atherosclerotic plaque [35].

There are several limitations of the present study. First is that the food frequency questionnaire used in ARIC tends to underestimate the absolute dietary intake for a particular nutrient, as is commonly the case for semi-quantitative dietary assessment tools [15]. Nevertheless, the FFQ used in this study was designed to rank participants and it is likely that we properly discriminated individuals in the highest and lowest categories of intake which was the focus of our analyses. The foods with the highest content in choline – eggs, milk, liver, red meat, poultry and fish, as well as the foods with the highest content in betaine – spinach, white bread and breakfast cereals, were items included in the ARIC FFQ. One can overcome the limitations of a dietary tool through the use of a biomarker. However, there is no reliable blood biomarker for dietary intake of choline. As it is known from clinical settings, plasma concentrations of choline and betaine decrease when subjects are fed a low choline diet, but the amount of decrease is not highly correlated with susceptibility to develop organ dysfunction while on this diet [36]. Another limitation of our investigation is the absence of supplemental B vitamin information including folate, which was queried only during subsequent examinations of the ARIC cohort.

Despite these limitations, there are a series of advantages for this study. The analyses are based on an extended follow-up of one of the largest biracial populations of U.S. adults, with the added strengths of validated CHD outcomes and a standardized collection of covariate information. These are elements that support the internal validity of the findings. Because in ARIC the dietary assessment was conducted before the mandatory supplementation of some foods (such as flour) with folate in the late 1990s, the study was able to minimize the interference with those compounds in its ascertainment of the exposure.

## Conclusion

Our study is the first to investigate the relationship between dietary intake of choline and choline plus betaine and the risk of incident coronary hearth disease in a large biethnic prospective cohort with participants of both genders. It represents an early investigation of two nutrients that have recently become available in our food composition database. We found that choline and choline plus betaine intakes were not predictors of incident CHD in the ARIC cohort. A higher choline intake did not prove protective for incident CHD among those with a low folate intake. Our findings offer information toward an understanding of the complex etiology of coronary occlusive events in relation to methyl-donor compounds. This study invites similar investigations in other populations and of other atherosclerotic events.

## Competing interests

The author(s) declare that they have no competing interests.

## Authors' contributions

AB, LEC and GH conceived of and designed the study. AB and LEC performed the statistical analyses. AB, LEC, AMSR and GH interpreted the results. AB drafted the manuscript. All authors revised the manuscript for intellectual content, and read and approved the final manuscript.

## Pre-publication history

The pre-publication history for this paper can be accessed here:


